# Deaths in New York

**Published:** 1876-07

**Authors:** 


### DEATHS IN NEW YORK.

*More* than one half of all the deaths in New York, during 1875. were of children under three years of age!

Twenty-three per cent, or nearly one-fourth of all who died, perished from some form of diseases affecting the throat and lungs.

All these diseases of the throat and lungs are brought on by habits of life, which owe themselves to our ignorance, or our recklessness, and consequently are avoidable diseases.

